# Non-human *lnc-DC* orthologs encode Wdnm1-like protein

**DOI:** 10.12688/f1000research.4711.2

**Published:** 2014-09-30

**Authors:** Johannes M. Dijkstra, Keith T. Ballingall

**Affiliations:** 1 Institute for Comprehensive Medical Science, Fujita Health University, Toyoake, 470-1192, Japan; 2 Moredun Research Institute, Penicuik, Midlothian, Scotland, EH26 0PZ, UK

## Abstract

In a recent publication in *Science*, Wang *et al*. found a long noncoding RNA (lncRNA) expressed in human dendritic cells (DC), which they designated *lnc-DC*. Based on lentivirus-mediated RNA interference (RNAi) experiments in human and murine systems, they concluded that *lnc-DC* is important in differentiation of monocytes into DC. However, Wang *et al*. did not mention that their so-called “mouse *lnc-DC ortholog*” gene was already designated “ *Wdnm1-like*” and is known to encode a small secreted protein.  We found that incapacitation of the *Wdnm1-like* open reading frame (ORF) is very rare among mammals, with all investigated primates except for hominids having an intact ORF. The null-hypothesis by Wang et al. therefore should have been that the human *lnc-DC* transcript might only represent a non-functional relatively young evolutionary remnant of a protein coding locus.  Whether this null-hypothesis can be rejected by the experimental data presented by Wang *et al*. depends in part on the possible off-target (immunogenic or otherwise) effects of their RNAi procedures, which were not exhaustive in regard to the number of analyzed RNAi sequences and control sequences.  If, however, the conclusions by Wang *et al*. on their human model are correct, and they may be, current knowledge regarding the *Wdnm1-like* locus suggests an intriguing combination of different functions mediated by transcript and protein in the maturation of several cell types at some point in evolution. We feel that the article by Wang *et al*. tends to be misleading without the discussion presented here.

## Correspondence

In their recent publication in *Science*, Wang *et al.* ^1^ aimed to identify lncRNAs involved in DC differentiation and function. In order to do this they used an established model of human DC differentiation from peripheral blood monocytes (Mo), based on addition of recombinant cytokines. They found that transcription of the human *Wdnm1-like* pseudogene ( *Wdnm1-like-ψ*), or *lnc-DC* as they call it, was robustly induced by the Mo-DC differentiation process. Furthermore, they found *Wdnm1-like-ψ* highly transcribed in other dendritic cells, and confirmed correlation of *Wdnm1-like-ψ* transcription with DC differentiation in several ways. To investigate a functional role of *Wdnm1-like-ψ* in their Mo-DC differentiation model, they used a lentivirus-mediated RNA interference (RNAi) system. The RNAi interference with *Wdnm1-like-ψ* fragments resulted in a pronounced effect on Mo-DC differentiation as measured by expression of genes and molecules involved in the immune system, the ability to take up antigen, and the capacity to stimulate T-helper cells. Wang *et al.* showed by a number of experiments that the *Wdnm1-like-ψ* transcript, in particular the 3’-end, has some specificity for binding to the STAT3 transcription factor and can reduce STAT3 dephosphorylation by phosphatase SHP1. And, importantly, they showed that in their human Mo-DC differentiation model the effect of STAT3 inhibition caused similar effects as knockdown of *Wdnm1-like-ψ*. They therefore postulated that human *Wdnm1-like-ψ* transcript is an important regulator of DC differentiation by enhancing STAT3 activity through prevention of STAT3 dephosphorylation by SHP1. The results and human model presented by Wang *et al.* are generally convincing, yet some questions remain, such as to why not for all experiments both “no transfection control” (used in a few experiments) and “control RNAi” (used in all experiments) were included, and why they only used a single RNAi control sequence. RNAi control sequences are relevant because off-target genes might be knocked down (e.g. Jackson *et al.* ^3^), but also because the lentivirus system using short hairpin RNA (shRNA) can have immunogenic properties in an shRNA-sequence-dependent manner (e.g. Kenworthy *et al.* ^4^). Notably, in some experiments Wang *et al.* ^1^ independently knocked down two different fragments of *Wdnm1-like-ψ*, with similar experimental results, thus reducing the chance that off-target effects of their RNAi systems influenced their conclusions. On the other hand, since the use of two positive RNAi systems suggests that Wang *et al.* were aware of the potential weaknesses of the system, this raises the question as to why they only used a single sequence for their RNAi control experiments. Regardless, we consider the part of their manuscript on human *Wdnm1-like-ψ* to be mostly solid and interesting, and the main reason why we are so (overly) critical is that acceptance of the model for human *Wdnm1-like-ψ* function as proposed by Wang *et al.* leads to a quite spectacular evolutionary model, as outlined below. Our view, which is supported in the accompanying referee report provided by Dr. Burchard, is that such a spectacular claim requires very robust evidence which in this case probably requires a higher number of RNAi controls.

**Figure 1.**
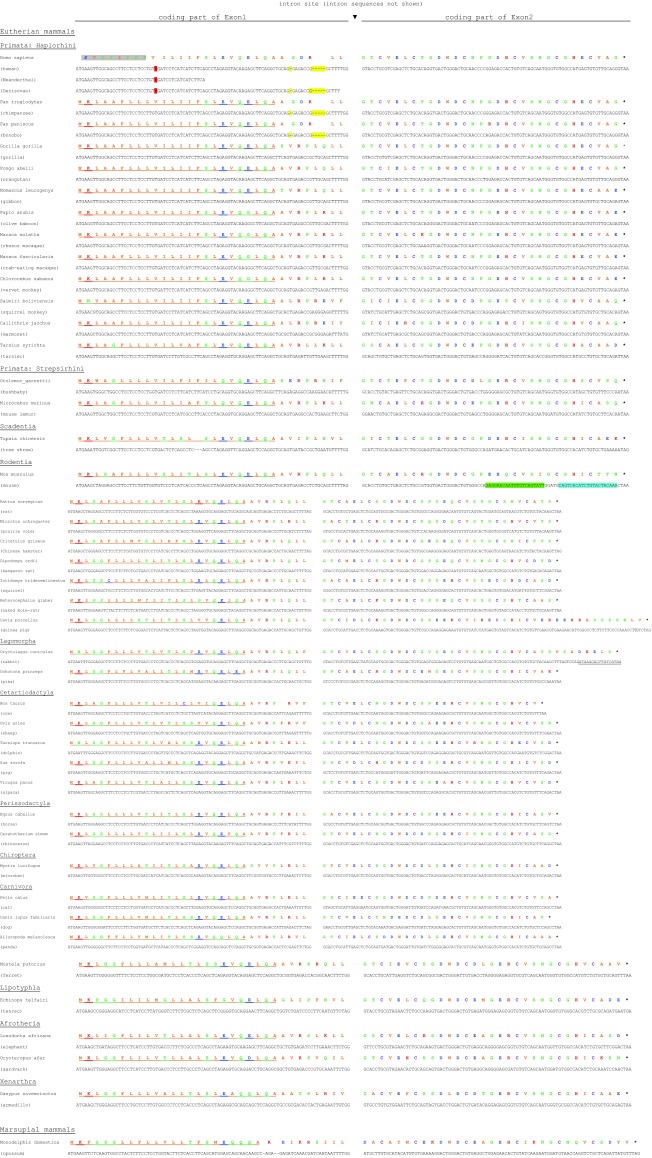
The highly conserved coding sequences of mammalian *Wdnm1-like*. The figure shows deduced Wdnm1-like amino acid sequences plus their coding nucleotide sequences in representative mammals. After evolutionary separation from gorilla, in an ancestor common to the genera *Pan* (including chimpanzee and bonobo) and *Homo* (including human, Neanderthal and Denisovan), the nucleotide region coding the N-terminus of the mature Wdnm1-like protein was modified by deletions (yellow shading). Nevertheless, in the genus *Pan* the *Wdnm1-like* open reading frame (ORF) remained intact. Only in *Homo* the Wdnm1-like coding sequence was interrupted by a frameshift through a single nucleotide deletion (red shading) within the leader peptide coding region (the resulting change in amino acids is shaded grey). For the human *Wdnm1-like* locus several transcripts (splicoforms) were found (Ensembl reports ENST00000590346, ENST00000588180, ENST00000587298, ENST00000590012, ENST00000589987, ENST00000592556, ENST00000566140, and ENST00000589777); however, we agree with Wang *et al.* ^1^ that software investigation of the known transcripts suggests that the human *Wdnm1-like* locus does not code a functional protein (analyses not shown). The marsupial *Monodelphis domestica* (opossum) was the only non-eutherian mammal for which we could identify *Wdnm1-like*, situated upstream of the gene *HEAT Repeat Containing 6* ( *HEATR6*) like its ortholog in eutherian mammals. To avoid gaps in the bulk of the figure, the N-terminus of the opossum sequence is not perfectly aligned with Wdnm1-like of eutherian mammals. Except for rabbit (see Methods section), the figure shows the ORFs of sequences corresponding to the murine Wdnm1-like protein coding transcript of NCBI accession NM_183249, while other (possible) splicoforms are neglected. The intron site is indicated by a downward triangle. Intron sequences are not shown, but the below listed genomic sequence reports agree with GT-AG borders. For most of the species, the depicted sequences were supported by transcript reports, as exemplified per species in the Methods section. In the figure, dashes indicate gaps that were introduced for optimal sequence alignment. The alignments were performed by hand. Amino acid sequences are indicated above the second nucleotides of codons. Basic residues are indicated in red, acidic residues in blue, and green residues are more hydrophilic than the orange ones (following reference ^9^). Cysteines are in violet. Asterisks correspond with stop codons. Predicted leader sequences are underlined. The mouse *Wdnm1-like* sequence was designated “mouse lnc-DC ortholog” by Wang *et al.* ^1^, and they targeted the regions shaded blue and green for transcript knockdown by “RNAi-1” and “RNAi-2”, respectively, using a lentivirus-mediated RNA interference system.

Whereas the presentation of their human data appears to be mostly correct, we feel that the way Wang *et al.* ^1^ present their mouse model is inappropriate. Wang *et al.* used a mouse model to confirm that knockdown of *Wdnm1-like(-ψ)* results in impaired DC differentiation. Technically these experiments in mice worked as they expected, indicated both by *in vitro* and *in vivo* results, and they also found that knockdown of murine *Wdnm1-like* could lead to reduction of STAT3 phosphorylation, although they apparently did not check if murine *Wdnm1-like* transcript can bind STAT3. However, even though Wang *et al.* refer to Gene symbol 110000G20Rik which mentions “Wdnm1-like”, they only present the readers with the term “mouse lnc-DC ortholog”. This is highly misleading as it suggests that the transcript also relates to a long noncoding RNA in mice. The authors even state “ *Taken together, our data suggest that lnc-DC is vital for DC differentiation in both human and mice*”. However, in mice the gene encodes a functional Wdnm1-like protein, as shown by recombinant analysis ^2^, and our extensive analysis of mammalian sequence databases indicates that the *Wdnm1-like* ORF incapacitation is very rare among mammals. Actually, among the eutherian mammals that we investigated and for which the relevant genomic region information was available, only humans (and Neanderthals and Denisovans) lacked the capacity to encode the otherwise highly conserved Wdnm1-like protein sequence ( Figure 1). At the level of the genus *Pan* (chimpanzee and bonobo) the N-terminus of the predicted mature protein differs from consensus, but even in gorilla and orangutan the encoded Wdnm1-like protein appears fully normal. So possibly the function of the Wdnm1-like protein started to lose importance after separation of *Homo*/ *Pan* from the other apes, which is quite recent in evolutionary terms. Calculation of synonymous (ds) versus nonsynonymous (dn) nucleotide substitution rates, using software available at http://www.hiv.lanl.gov/content/sequence/SNAP/SNAP.html, indicates conservation of Wdnm1-like protein function after most of the animals shown in Figure 1 had separated in evolution. Namely, in pairwise comparisons, for the depicted set of eutherian mammals except *Pan*/ *Homo* the average ds/dn ratio is 3.5, and for the set of primates except *Pan*/ *Homo* this value is 3.0. Thus, although in each individual species experimental evidence would still be required, it is expected that most eutherian mammals possess functional Wdnm1-like protein. Probably because of lack of directed investigations, naturally expressed endogenous full-size Wdnm1-like proteins have not yet been reported. However, our search of the PeptideAtlas database of peptides identified by mass spectrometry identified a rat ( *Rattus Norwegicus*) Wdnm1-like fragment encoded by properly spliced *Wdnm1-like* transcript ( http://www.peptideatlas.org, peptide PAp03984316).

The name *Wdnm1-like* was first coined by Adachi *et al.* ^5^, who found that *Wdnm1-like* transcript was differentially expressed in limbal versus central corneal epithelia in rat, and who observed similarity of the encoded protein with Wdnm1. Within the serial analysis of gene expression (SAGE) experiment by Adachi *et al.* ^5^, *Wdnm1-like* comprised the most abundant SAGE tag present exclusively in the limbal library, and the authors hypothesized that Wdnm1-like might be a marker of limbal stem cells. They could, however, not rule out the possibility that Wdnm1-like was expressed by other cell types present in limbal epithelia, such as for example dendritic cells. A later study on rodent *Wdnm1-like* was performed in mice by Wu and Smas ^2^. Wu and Smas got interested in *Wdnm1-like* after they found it highly upregulated upon differentiation of preadipocytes into adipocytes. They found *Wdnm1-like* to be selectively expressed in liver and adipose tissue, and enriched in white adipose depots versus brown. Recombinant expression of tagged murine Wdnm1-like in HT1080 human fibrosarcoma cells revealed a small secreted protein ^2^. Because Wdnm1-like is a distant member of the whey acidic protein/four-disulfide core (WAP/4-DSC) family, of which several members have roles as proteinase inhibitors, Wu and Smas speculated that Wdnm1-like might have a similar function. An important class of extracellular proteases involved in adipocyte differentiation are the matrix metalloproteinases (MMPs), which can degrade extracellular matrix (ECM) components. Therefore, Wu and Smas investigated whether MMPs expressed by HT1080 were affected by the recombinant Wdnm1-like expression, and they found an increased amount of the active form of MMP-2 ^2^. Thus, rather than having an inhibitory effect, Wdnm1-like appears to enhance activation of a protease. Wu and Smas conclude with “ *Future studies are required to address the mechanism(s) underlying the function and regulation of adipocyte-secreted Wdnm1-like*” ^2^, and according to literature this situation has not changed since then.

Looking at the combined publications, a very complicated picture emerges. In most mammals the *Wdnm1-like* locus encodes a protein, with humans as an exception which is possibly unique. In rat, *Wdnm1-like* is differentially expressed in limbal versus central corneal epithelia ^4^. In mouse, *Wdnm1-like* is expressed upon adipogenesis, and Wdnm1-like protein enhances the production of active MMP-2 ^2^. In human and mouse, the *Wdnm1-like(-ψ*) transcript appears functionally associated with dendritic cell differentiation, and at least in humans this may be mediated by binding of the transcript to STAT3 ^1^. This leaves questions for future research such as, for example, whether human *Wdnm1-like-ψ* transcript is also associated with adipogenesis, and whether murine *Wdnm1-like* transcript exerts its function on DC differentiation by binding to STAT3 or by encoding Wdnm1-like protein. Supporting that the Wdnm1-like proteins and transcripts in some extinct or extant animals may have (had) synergetic functions, is the fact that differentiation of both adipocytes and limbal epithelial cells can involve STAT3 ^6, 7^. So, despite our points of criticism, we think that the results and human model by Wang *et al.* may be valid and part of a more complex evolutionary scenario that involves distinct functions at the transcript and protein level, and a number of different tissues and cell types. In general, we think that studies on long noncoding RNAs typically require discussion of the evolutionary context ^8^, especially when dealing with wide species borders such as between human and mouse.

### Additional note 1

A nice speculation allowed by the combined referenced articles is that Wdnm1-like protein might promote Mo-DC differentiation in humans. After all, murine Wdnm1-like protein was found to enhance MMP-2 activity of human HT1080 cells ^2^, concluding that humans did not lose their sensitivity to Wdnm1-like protein.

### Additional note 2

We did not feel comfortable with the amount of space and visibility the editors of the journal *Science* were able to offer us via their commenting mechanism for the discussion presented here. Therefore, we declined their offer, and instead deemed publication in *F1000Research* a more appropriate vehicle. Through F1000Research we asked specialists and the corresponding authors of several of the referenced articles, including the article by Wang *et al.*, to provide referee reports (which may also include broad views) on our discussion. We are pleased with the excellent referee reports received from Dr. Smas and Dr. Ren, and Dr. Burchard, and recommend them to the readers of our article. Importantly, Drs. Smas and Ren confirmed that we correctly summarized reports on rodent *Wdnm1-like* and listed additional evidence to that matter, while Dr. Burchard substantiated and detailed our notion that the RNAi experiments by Wang *et al.* were inconsistent and probably incomplete. We would welcome comments from the group of Wang *et al.* and also encourage other researchers to leave comments.

### Additional note 3

Wdnm1-like protein appears to be very interesting. Not only may it be involved in differentiation of several cell types, it also is intriguing because it appears highly conserved throughout eutherian mammals and (rather) uniquely lost in hominids. It may help determine what makes us human.

## Methods

The partial *Wdnm1-like* sequence information available for extinct hominids, namely, for Neanderthal and Denisovan, was retrieved using the UCSC genome browser ( http://genome.ucsc.edu). All other sequences shown in the figure were retrieved from Ensembl ( www.ensembl.org/) or NCBI ( http://www.ncbi.nlm.nih.gov/) databases. For a representative list of model species, we investigated database sequences of all mammals for which genomic sequences are available in the Ensembl database, and also of *Pan paniscus* (bonobo). For some of those animals sequence information for the *Wdnm1-like* ORF or for its expected genomic site was incomplete, and in such case the sequence is not included in the alignment figure.

Leader peptide sequences were predicted by SignalP software ( www.cbs.dtu.dk/services/SignalP/) and are underlined. For the species *Panio anubis* (olive baboon), *Heterocephalus glaber* (naked mole-rat), *Myotis lucifugus* (microbat), and *Dasypus novemcintus* (armadillo), besides the here indicated evolutionary conserved cleavage site, the SignalP software also predicted an alternative cleavage site with a calculated higher likelihood (not shown).

**Species-specific information related to sequences depicted in Figure 1:**

### *Homo sapiens* (human)

The depicted human *Wdnm1-like* pseudogene sequence maps within Ensembl database GRCh37, Chr.17 positions 58162470-to-58165647, reverse orientation. Furthermore, the depicted sequence corresponds with positions 182-to-366 of the transcript sequence of NCBI accession NR_030732.1.

### *Homo sapiens* (Neanderthal) (whether Neanderthal should be considered a subspecies of *Homo sapiens* is a matter of debate)

The depicted Neanderthal sequence was identified from genomic DNA fragments (Read names: M_SL-XAT_0004_FC30PMDAAXX:1:87:384:343, M_BIOLAB29_Run_PE51_1:2:9:981:262 and C_M_SOLEXA-GA04_JK_PE_SL21:8:99:944:526) isolated from the Vi33.16 and Vi33.25 Neanderthal samples ^10^ using the UCSC genome browser by comparison with the human Wdnm1-like sequence. The depicted sequence fragment is identical to that of human.

### *Homo sapiens* (Denisovan) (whether Denisovan should be considered a subspecies of *Homo sapiens* is a matter of debate)

The depicted Denisovan sequence was obtained as described for the Neanderthal sequences, and corresponds to part of the read M_SOLEXA-GA02_00040_PEdi_MM_3:8:112:19220:10730#AACCATG,CTCGATG (Meyer *et al.* ^11^). The depicted sequence fragment is identical to that of human.

### *Pan troglodytes* (chimpanzee)

The depicted chimpanzee *Wdnm1-like* sequence maps within Ensembl database CHIMP2.1.4, Chr.17 positions 588018940-to-58805072, reverse orientation. Transcription is supported by NCBI SRA database sequence reports, such as for example gnl|SRA|DRR003370.54864751.1 of experiment set DRX002694.

### *Pan paniscus* (bonobo, or pygmy chimpanzee)

The depicted bonobo *Wdnm1-like* sequence maps within the genomic sequence of NCBI database accession gb|AJFE01016111.1|, positions 11414-to-14585, forward orientation. Transcription is supported by NCBI SRA database sequence reports, such as for example gnl|SRA|SRR873628.59588401.2 of experiment set SRX290737.

### *Gorilla gorilla* (gorilla)

The depicted gorilla *Wdnm1-like* sequence maps within Ensembl database gorGor3.1, Chr.5 positions 23775798-to-23778981, forward orientation. Transcription is supported by NCBI SRA database sequence reports, such as for example gnl|SRA|SRR306801.5146816.1 of experiment set SRX081945.

### *Pongo abelii* (Sumatran orangutan)

The depicted orangutan *Wdnm1-like* sequence maps within Ensembl database PPYG2, Chr.17 32711864-to-32715478, forward orientation. This is a recent intrachromosomal duplication of the original *Wdnm1-like* gene. The Ensembl database shows that Sumatran orangutan still has at least part of that original *Wdnm1-like* gene upstream of *HEATR6*, but information of that region is incomplete. Evidence for transcription of *Wdnm1-like* in orangutan is provided by NCBI SRA database sequence reports *for Pongo pygmaeus* (Bornean orangutan), such as for example gnl|SRA|SRR306799.12707499.1 of experiment set SRX081943.

### *Nomascus leucogenys* (gibbon)

The depicted gibbon *Wdnm1-like* sequence maps within the genomic sequence of NCBI database accession gb|ADFV01146912.1|, positions 1414-to-4561, reverse orientation.

### *Papio anubis* (olive baboon)

The depicted olive *Wdnm1-like* sequence maps within Ensembl database Panu_2.0, scaffold JH685681 positions 60156-to-64601, reverse orientation. Transcription is supported by NCBI SRA database sequence reports, such as for example gnl|SRA|SRR1045089.118535973.1 of experiment set SRR1045089.

### *Macaca mulatta* (rhesus macaque)

The depicted rhesus macaque *Wdnm1-like* sequence maps within Ensembl database MMUL_1, scaffold 1099548049739 positions 121534-to-124737, forward orientation. Transcription is supported by NCBI SRA database sequence reports, such as for example gnl|SRA|SRR1240160.28991243.2 of experiment set SRR1240160.

### *Macaca fascicularis* (crab-eating macaque)

The depicted crab-eating macaque *Wdnm1-like* sequence maps within the genomic sequence of NCBI database accession gb|AEHL01027073.1|, positions 5255-to-8524, forward orientation. Transcription is supported by NCBI SRA database sequence reports, such as for example gnl|SRA|DRR001354.3296367.1 of experiment set DRX000951.

### *Chlorocebus sabaeus* (vervet monkey)

The depicted *Wdnm1-like* sequence maps within Ensembl database ChlSab1.0, Chr.16 positions 29807079-to-29810262, reverse orientation. Transcription is supported by NCBI SRA database sequence reports for the closely related species *Chlorocebus aethiops* (green monkey), such as for example gnl|SRA|SRR1178509.592424.2 of experiment set SRR1178509.

### *Saimiri boliviensis* (Bolivian squirrel monkey)

The depicted squirrel monkey *Wdnm1-like* sequence maps within Ensembl database SalBol1.0, scaffold JH378137 positions 636410-to-639575, forward orientation. Transcription is supported by NCBI SRA database sequence reports, such as for example gnl|SRA|SRR500949.3269772.2 of experiment set SRX149650.

### *Callithrix jacchus* (marmoset)

The depicted marmoset *Wdnm1-like* sequence maps within Ensembl database C_jacchus3.2.1, Chr.5 positions 88345809-to-88348914, reverse orientation. Transcription is supported by NCBI database accession gb|GAMR01043615.1|.

### *Tarsius syrichta* (tarsier)

The depicted tarsier *Wdnm1-like* sequence maps within Ensembl database tarSyr1, scaffold_1716 positions 51738-to-55873, reverse orientation.

### *Otolemur_garnettii* (bushbaby)

The depicted bushbaby *Wdnm1-like* sequence maps within Ensembl database OtoGar3, scaffold GL873613 positions 7509108-to-7514627, reverse orientation.

### *Microcebus murinus* (mouse lemur)

The depicted mouse lemur *Wdnm1-like* sequence maps within Ensembl database micMur1, GeneScaffold_1067 positions 49762-to-53887, reverse orientation. Transcription is supported by NCBI SRA database sequence reports, such as for example gnl|SRA|SRR832933.720157201.1 of experiment set SRX270644.

### *Tupaia chinensis* (Chinese tree shrew)

The depicted Chinese tree shrew *Wdnm1-like* sequence maps within Ensembl database TREESHREW, scaffold_15853 positions 2941-to-6216, reverse orientation. Transcription is supported by NCBI SRA database sequence reports, such as for example gnl|SRA|SRR518934.53798716.1 of experiment set SRX157966.

### *Mus musculus* (mouse)

The depicted mouse *Wdnm1-like* sequence maps within Ensembl database GRCm38, Chr.11 positions 83747027-to-83749327, forward orientation. Transcription is supported by for example NCBI accession NM_183249.1.

### *Rattus norvegicus* (rat)

The depicted rat *Wdnm1-like* sequence maps within Ensembl database Rnor_5.0, Chr.10 positions 70671110-to-70673427, forward orientation. Transcription is supported by for example NCBI accession gb|EF122001.1|.

### *Microtus ochrogaster* (prairie vole)

The depicted prairie vole *Wdnm1-like* sequence maps within Ensembl database MicOch1.0, Chr.7 positions 15310620-to-15312860, reverse orientation. According to the Ensembl database the prairie vole also has an intronless copy of *Wdnm1-like* gene on Chr.X (not shown). Transcription is supported by NCBI SRA database sequence reports, such as for example gnl|SRA|SRR058428.108679.2 of experiment set SRX018513.

### *Cricetulus griseus* (Chinese hamster)

The depicted hamster *Wdnm1-like* sequence maps within the genomic sequence of NCBI database accession gb|AMDS01007412.1|, positions 15363-to-17750, forward orientation.

### *Dipodomys ordii* (kangaroo rat)

The depicted kangaroo rat *Wdnm1-like* sequence maps within Ensembl database dipOrd1, scaffold_2778 positions 48464-to-52516, reverse orientation.

### *Ictidomys tridecemlineatus* (thirteen-lined ground squirrel)

The depicted squirrel *Wdnm1-like* sequence maps within Ensembl database spetri2, scaffold JH393300 positions 533158-to-536139, forward orientation.

### *Heterocephalus glaber* (naked mole-rat)

The depicted naked mole-rat *Wdnm1-like* sequence maps within Ensembl database HetGla_female_1.0, scaffold JH602188 positions 3555009-to-3557720, forward orientation.

### *Cavia porcellus* (domestic guinea pig)

The depicted guinea pig *Wdnm1-like* sequence maps within Ensembl database cavPor3, scaffold_32 positions 10788821-to-10791159, reverse orientation.

### *Oryctolagus cuniculus* (rabbit)

The depicted rabbit *Wdnm1-like* sequence maps within Ensembl database OryCun2.0, Chr.19 positions 25079843-to-25084775, forward orientation. The underlined part in Italic font at the 3’end belongs to a third exon. Transcription is supported by for example NCBI accession gb|GBCH01008538.1|.

### *Ochotona princeps* (pika)

The depicted pika *Wdnm1-like* sequence maps within Ensembl database OchPri3, scaffold JH802106 positions 113719-to-116807, forward orientation. Transcription is supported by NCBI SRA database sequence reports, such as for example gnl|SRA|SRR850200.108627.2 of experiment set SRX277346.

### *Bos taurus* (cattle)

The depicted cattle *Wdnm1-like* sequence maps within Ensembl database UMD3.1, Chr.19 positions 14485956-to-14490393, reverse orientation. Transcription is supported by for example NCBI accession gb|AW484602.1|.

### *Ovis aries* (sheep)

The depicted sheep *Wdnm1-like* sequence maps within Ensembl database Oar_v3.1, Chr.11 positions 13759463-to-13763966, reverse orientation. Transcription is supported by for example NCBI accession gb|CK830678.1|.

### *Tursiops truncatus* (dolphin)

The depicted dolphin *Wdnm1-like* sequence maps within the genomic sequence of NCBI database accession gb|ABRN02348024.1|, positions 2742-to-5945, forward orientation.

### *Sus scrofa* (pig)

The depicted pig *Wdnm1-like* sequence maps within the genomic sequence of NCBI database accession gb|AJKK01193461.1|, positions 3786-to-7100, reverse orientation. Transcription is supported by for example NCBI accession dbj|AK399701.1|.

### *Vicugna pacos* (alpaca)

The depicted alpaca *Wdnm1-like* sequence maps within Ensembl database vicPac1, GeneScaffold_1352 positions 716864-to-719675, reverse orientation.

### *Equus caballus* (horse)

The depicted horse Wdnm1-like sequence maps within Ensembl database EquCab2, Chr.11 positions 36986601-to-36989473, reverse orientation. The Ensembl database indicates additional copies of *Wdnm1-like* on Chr.11 (not shown). Transcription is supported by for example NCBI accession gb|DN508620.1|.

### *Ceratotherium simum* (rhinoceros)

The depicted rhinoceros *Wdnm1-like* sequence maps within Ensembl database CerSimSim1, scaffold JH767772 positions 17445128-to-17447968, reverse orientation.

### *Myotis lucifugus* (microbat)

The depicted microbat *Wdnm1-like* sequence maps within Ensembl database Myoluc2.0, scaffold_GL430154 positions 92198-to-94525, reverse orientation.

Transcription is supported by NCBI SRA database sequence reports, such as for example gnl|SRA|SRR1013468.27145136.1 of experiment set SRR1013468.

### *Felis catus* (cat)

The depicted cat *Wdnm1-like* sequence maps within the genomic sequence of NCBI database accession gb|AANG02057756.1|, positions 9507-to-13123, forward orientation. Transcription is supported by NCBI SRA database sequence reports, such as for example gnl|SRA|SRR835496.27404932.1 of experiment set SRX272142.

### *Canis lupus familiaris* (dog)

The depicted dog *Wdnm1-like* sequence maps within Ensembl database CanFam3.1, Chr.9 positions 37619501-to-37622242, reverse orientation. Transcription is supported by for example NCBI accession gb|DR107055.1|.

### *Ailuropoda melanoleuca* (panda)

The depicted panda *Wdnm1-like* sequence maps within Ensembl database ailMel1, scaffold GL193203 positions 54404-to-57462, forward orientation.

### *Mustela putorius* (ferret)

The depicted ferret *Wdnm1-like* sequence maps within Ensembl database MusPutFur1.0, scaffold GL896917 positions 9435086-to-9438171, forward orientation. Transcription is supported by for example NCBI accession gb|JR792458.1|.

### *Echinops telfairi* (lesser hedgehog tenrec)

The depicted tenrec *Wdnm1-like* sequence maps within the genomic sequence of NCBI database accession gb|AAIY02150441.1|, positions 1061-to-5393, forward orientation.

### *Loxodonta africana* (elephant)

The depicted elephant *Wdnm1-like* sequence maps within Ensembl database loxAfr3, scaffold_31 positions 5685863-to-5689773, forward orientation. Transcription is supported by NCBI SRA database sequence reports, such as for example gnl|SRA|SRR1041765.37646273.1 of experiment set SRR1041765.

### *Orycteropus afer* (aardvark)

The depicted aardvark *Wdnm1-like* sequence maps within Ensembl database OryAfe1, scaffold JH863914 positions 5948889-to-5951515, reverse orientation.

### *Dasypus novemcinctus* (nine-banded armadillo)

The depicted armadillo *Wdnm1-like* sequence maps within Ensembl database Dasnov3.0, scaffold JH562945 positions 1888971-to-1892017, forward orientation. Transcription is supported by NCBI SRA database sequence reports, such as for example gnl|SRA|SRR494776.6845635.2 of experiment set SRX146634.

### *Monodelphis domestica* (opossum)

The depicted opossum *Wdnm1-like* sequence maps within Ensembl database BROADO5, Chr.2 positions 498828348-to-498830609, reverse orientation. Transcription is supported by NCBI SRA database sequence reports, such as for example gnl|SRA|SRR908062.57922637.2, gnl|SRA|SRR873400.62402918.1, gnl|SRA|SRR943348.21681365, gnl|SRA|SRR943348.11424624, and gnl|SRA|SRR943348.9801988 of experiment sets SRX310006 and SRX290643 (because other *Wdnm1-like* transcript information appears lacking for marsupials, we here provide SRA database accessions that together cover the full ORF).
