# Supplementary material for: Non-human lnc-DC orthologs encode Wdnm1-like protein
Source: F1000Res. 2014 Sep 30;3:160. Originally published 2014 Jul 11. [Version 2] doi: 10.12688/f1000research.4711.2 (PMC4184305; doi:10.12688/f1000research.4711.2)
Supplement: Peer review history

# Peer review: Referee response for version 1

**DOI:** 10.5256/f1000research.5033.r6091

**Published:** 2014-09-09

**Authors:** Julja Burchard

**Affiliations:** 1 Rosetta Inpharmatics LLC, a wholly-owned subsidiary of Merck and Co., Inc., Seattle, USA

This item of correspondence is in reference to Wang *et al.* (2014), concerning possible cytosolic protein-binding function in conventional dendritic cells by a human noncoding RNA and its mouse ortholog.  The correspondents note that the lncRNA designated “lnc-DC” by Wang *et al.* is a protein-coding transcript, Wfdc21 or Wdnm1-like, in all mammals with available sequence other than hominids ( *Homo sapiens sapiens*, *Neanderthal* and *Denisova*), and suggest that Wang *et al. *would have been well advised to discuss this information, as they propose functions for the transcript in human and mouse cells.

The correspondent authors provide an abbreviated summary of the original article in the abstract with suitable detail in the body of the letter.  The letter brings up an intriguing point worthy of discussion.  Drs. Dijkstra and Ballingall frame and present their observations well and do a thorough job assembling and aligning sequences in support.  To their point, recent work finds that only 0.3% of anthropoid-specific constrained sequences – functional primate innovations – are coding ( del Rosario *et al*., 2014).  If a similar proportion holds for significant functions hominids have ceased to perform, Wang *et al.* have identified an unusual case and have not highlighted it as such.

 A few items of follow-up may be of interest to clarify and extend the evolutionary observations in this correspondence.

1. Is the frameshift in the erstwhile signal peptide coding sequence in the human reference genome reproduced in all 1000-genomes data, or is WFDC21P a polymorphic human pseudogene?
2. Is guidance by the human reference sequence in assembly of *Neanderthal* and *Denisova* genomes a potential factor in their reproduction of the reported human frameshift?
3. Is there human genetic variation at this locus tied to variation in trait expression, or alternatively is there evidence that variation in this small gene is sufficiently suppressed to leave no functional variation for genetic association studies to mine?  Either could be consistent with a significant role in immune function as proposed by Wang *et al. *
4. What is found at the position syntenic to Wfdc21 in marsupials other than the one noted as sharing this gene, and in lower model organisms?   This may help clarify the nature of the apparent mammalian innovation at this locus.
5. Do regulatory elements for WFDC21P differ between hominids and other species?  If Wfdc21 is a gene with active RNA and protein products, their function will have evolved in the context of cell-type specific expression.  Fantom5 CAGE tag data suggests a difference in regulation of human WFDC21P vs. mouse Wfdc21.  Although parallel samples are not available for all tissues and cell types, mouse data show strongest expression in myeloid suppressor cells with significant expression also in liver and skin, while human data show 1000x lower maximum expression with best expression in migratory Langerhans cells.
6. Do the sections of WFDC21P-RNA highlighted as functional by Wang *et al. *show signs of hominid or mammalian constraint?  It is intriguing that Wang *et al. *show data suggesting STAT3 binding by the 3’-end of WFC21P-RNA in a section downstream of the ORF and thus potentially available for secondary function. 

The correspondent authors also discuss the technical merits of the work presented in Wang *et al.,* as indicated by the potential novelty of the findings.  Four additional points may be made here.

1. It is important to establish the RNA-dependence of activities discussed by Wang *et al*.  While the correspondents are satisfied that lack of human protein coding has been demonstrated, some experiments could be clearer.  Wfdc21 is a secreted protein.  Did Wang *et al.* examine the supernatant as well as the cells in which Flag-tagged fusions were expressed? 
2. The correspondents comment on the inconsistent use of controls.  Indeed, the sole controlled experiment suggesting RNA-protein association appears to be the pulldown of STAT3 with biotinylated WFDC21P RNA, with specific absence of the STAT3 band in the antisense control.  While RIP with STAT3 experimental and STAT1 control antibodies was conducted, no sequencing is reported so the specificity of interaction with STAT3 is not known.  Further, figures on RNA-FISH visualization of association of STAT3p with WFDC21P-RNA do not show controls. 
3. Wang *et al. *rely on inhibitors to demonstrate WFDC21P-RNA function.  As the correspondents note, one shRNA sequence is primarily employed and it is not consistently paired with varied on- or off-target controls.  Literature on functional siRNA screens suggests that a half dozen RNAi sequences with independent seeds are required for dissociation of off- and on-target activities.  Further, Wang *et al. *have performed expression profiling on shRNA-treated cells.  The profiles can be examined for seed-based off-target activity and for inflammatory response to the lentiviral vector according to published methods.  It will be important to establish whether the dendritic cell proteins whose differential expression is highlighted by Wang *et al. *show shRNA-matching seed sequences in their 3’UTRs or respond to lentiviral infection. 
4. Wang *et al.* also use published STAT3 inhibitors to elucidate the role of WFDC21P-RNA.  It would be intriguing to speculate that an RNA-protein interaction site helps to define the STAT3 binding site of published inhibitor S3I-201 and its effects on STAT3 activity.  However, the supplementary material provided by Wang *et al.* show much more profound effects on cytokine production by small molecules than shown for WFDC21P shRNA in the main paper, although effects on T-cell activation remain similar.

I have read this submission. I believe that I have an appropriate level of expertise to confirm that it is of an acceptable scientific standard.

# Response

**Published:** 2014-09-10

**Authors:** Johannes M. Dijkstra

**Affiliations:** Fujita Health University, Japan

Dear Dr. Burchard,

We thank you for your extensive and valuable comments. Like the comments by Drs. Smas and Ren, we embrace them as generally positive. Your comments add accuracy to our story, especially regarding the technical part of the RNA investigation by Wang and co-workers.

You wonder, as do Drs. Smas and Ren, and as do we, whether in some individuals or under some conditions, humans may express Wdnm1-like protein. After all, the sequence for the mature protein appears intact, and requirements for a leader peptide are not very unique. However, our rather extensive database investigations could not retrieve a human sequence expected to encode a functional Wdnm1-like protein. Identical frameshifts in Neanderthal and Denisovan *Wdnm1-like* sequence reports, for the reliability of which we have to depend on the respective authors, argue against the likeliness of functional/nonfunctional Wdnm1-like polymorphism in modern humans. An expressed sequence tag (EST), reported as GenBank accession CD692402, suggests that an individual from southern China may have a protein coding *Wdnm1-like* sequence; however, besides repair of the frame-shift in the leader coding region, this sequence has an additional unique modification, and the sequence report may contain technical errors. In short, we could not obtain evidence for intact Wdnm1-like coding sequences in humans, but cannot exclude the possibility that such sequences exist. We would welcome if anyone could provide such evidence or indications.

We took a brief look at Wdnm1-like evolution beyond eutherian mammals. However, except for the mentioned case in opossum, at this evolutionary distance it becomes difficult to distinguish Wdnm1-like orthologues from other family members, and it would become more a discussion on the evolution of Wdnm1-like plus related molecules than of Wdnm1-like alone. Because the primary goal of our study is the discussion of the Wang *et al.* article, which is confined to eutherian mammals, we feel a discussion of deeper Wdnm1-like evolution would make our study too complicated.

Although we did not make systematic comparisons among various genes, we feel that overall the 3’-end region of human *Wdnm1-like-* *y* transcript which is believed to interact with STAT3, is not especially well conserved among mammals. However, without knowing the precise sequence motif or RNA secondary structure important for that binding, we probably shouldn’t speculate on presence or absence of evolutionary constraints on that region.

You raise four additional points regarding the technical merits of the work presented by Wang and co-workers. Importantly, you agree with us that the Wang *et al. *study was inconsistent and probably incomplete in the use of controls. Some points you raise are valid speculations and questions, whereas others can be considered as criticism of the Wang *et al. *article. In our opinion that criticism is mostly right. However, we prefer not to change the open style of our technical comments, and hope that the readers will find the specific issues that you raise when reading your report.

Thank you again for your hard and valuable work.

Sincerely,

Johannes M. Dijkstra and Keith T. Ballingall

# Peer review: Referee response for version 1

**DOI:** 10.5256/f1000research.5033.r5905

**Published:** 2014-08-21

**Authors:** Cynthia Smas, Gang Ren

**Affiliations:** 1 Department of Biochemistry and Cancer Biology, University of Toledo, Toledo, OH, USA

This laboratory (Smas) published a manuscript on the *Wdnm1-like* murine gene in 2008 ^1^; as such we have been invited to review and comment here on the paper by Dijkstra and Ballingall. Their report presents extensive sequence analysis, for a range of species, of the *Wdnm1-like* gene. The title of the report is an accurate representation of the content of the article and the important points are well-summarized in the abstract. The extensive sequence information they provide in their text is in a format that allows the reader to reassess the sequence analysis data if so desired.

The information provided by Dijkstra and Ballingall makes it abundantly clear that the *Wdnm1-like* locus is predicted to contain a *bona fide* open reading frame that would encode a small secreted protein in all extant species examined except humans. This protein is Wdnm1-like, a member of the WFDC (Whey Acidic Protein Four Disulfide Core) protein family.  In all but hominids, the predicted protein encoded by the *Wdnm1-like* gene, also recently annotated in the Gene NCBI database as *Wfdc21*, has clear homology with the WFDC protein family ^2^. In hominids ( *Homo sapiens*) this locus contains the Wdnm1-like pseudogene, which Wang and co-authors report encodes a long non-coding RNA that they have named *lnc-DC*.  In humans, a one base pair deletion is present near the start of what would have been the open reading frame for the Wdnm1-like protein. This frame shift apparently eliminates the protein coding ability of the human gene. However, restoration of the correct open reading frame would be predicted to generate an encoded protein with significant homology to the murine Wdnm1-like protein.

The detailed sequence analyses and other information provided by Dijkstra and Ballingall  is valuable to the long-noncoding RNA research community and to those following the recent research into dendritic cell differentiation, in respect to the *Science *report  that was published in April 2014 by Wang and co-authors “ *The STAT3-Binding Long Noncoding RNA lnc-DC Controls Human Dendritic Cell Differentiation*” ^3^.  In regard to the *Science* report by Wang and co-authors the design, methods and analysis of the results in the paper by Dijkstra and Ballingall are well-explained and are they appropriate to the topic. The conclusions they reach are sensible, generally balanced and justified. In a few instances, however, the tone of the writing is a bit aggressive. As further discussed below, it appears an assumption is made by Dijkstra and Ballingall that Wang and co-authors were aware of the distinction that the human locus encodes a pseudogene, while in mouse this locus encodes the Wdnm1-like protein.

This laboratory (Smas) identified murine *Wdnm1-like* as an adipogenesis-induced gene in a report in 2008, wherein a role for Wdnm1-like protein in modulating MMP activity was also reported. Shortly after this publication in 2008, when considering generation and study of null mice for *Wdnm1-like*, it became apparent that the human locus had a single base pair deletion early in an otherwise predicted open reading frame. Therefore, it likely encoded a pseudogene in humans and not the Wdnm1-like protein.  Given this, it was decided that this laboratory (Smas) would not go further on the project in respect to the study of the Wdnm1-like protein. As such, the human gene or transcript has not been addressed in studies from this laboratory (Smas), and we not aware of any data reporting on expression of this transcript in human adipose tissue.  However, while not the subject of such studies, murine Wdnm1-like has been mentioned within several publications on adipocytes and adipose tissue ^4^^-^^6^. These relate to the finding that the Wdnm1-like transcript is highly enriched in expression in white *vs.* brown murine adipocytes/adipose tissue ^4^^-^^6^. Of interest, knockout mice for Wfdc21 (Wfdc21 *^tm1a(KOMP)Wtsi^* ) are now available by embryo resuscitation through the KOMP project (Project ID: CSD49368) and would serve as a highly useful system in which to address the role of Wdnm1-like  in DC cell maturation in mice.

In the prior publication on Wdnm1-like from this laboratory (Smas), our studies utilized a murine Wdnm1-like expression construct with a C-terminal epitope tag, and cell transfection studies. These showed that a protein of predicted size for Wdnm1-like was ectopically expressed and secreted into culture media. Dijkstra and Ballingall do accurately describe our studies and clearly state that our work on Wdnm1-like utilized recombinant ectopic expression of the predicted murine Wdnm1-like open reading frame. However, one would have liked to have seen Dijkstra and Ballingall make it even more clear, earlier on in their text, that the endogenous Wdnm1-like  protein has not yet been demonstrated in any system/species.  A quick Google search fails to find an available antibody to the Wdnm1-like protein, so the endogenous protein remains to be investigated. 

The work by Dijkstra and Ballingall makes several very important points:

 

1. It provides well-needed clarification and extensive documentation that apparently only for hominids does the locus for *Wdnm1-like* encode a long non-coding RNA, while in all other species specifically examined (which was quite extensive) this locus contains an open reading frame for the Wdnm1-like protein.
2. It raises concerns as to the ultimate responsibility of Wang and co-authors to report the full range of information on such distinctions within their *Science* report, if they indeed were aware of such.  Only Wang and co-authors can inform us of their extent of knowledge of the protein coding nature of murine *vs.* human *Wdnm1-like* at the time of their *Science* publication. Thus it is not possible at this point to know whether such information was selectively omitted. But as Dijkstra and Ballingall point out, Wang and co-authors refer to the GenBank entry for murine Wdnm1-like , also known as 100001G20Rik and now formally named Wfdc21. This GenBank entry contains citations for publications on murine Wdnm1-like ^1^^,^^7^.  It seems very odd if Wang and co-authors were not aware of the distinctions between the human and murine forms of *Wdnm1-like*, particularly in today’s age of well-curated databases. In fact, the NCBI Unigene entry for this gene reveals the human version is annotated as *Wdnm1-like* pseudogene (LOC645638, Hs.463652). Perhaps this was one more instance of a research group “rediscovering and renaming” a gene that was previously published on. This is all too common of late; doing so essentially tosses aside the already peer-reviewed and published work of others ^1^^,^^7^.  However, until further clarification is forthcoming by Wang and co-authors, one would hope they would be provided with the benefit of the doubt on the facts and intentions in regard to this matter.
3. A third valid concern raised by Dijkstra and Ballingall, is whether the knowledge that the murine gene is predicted to be protein encoding, while the human gene encodes a long non-coding RNA ( *lnc-DC*), impacts the quality or interpretation of the data in the manuscript by Wang and co-authors. It appears that for the vast majority of the studies in their *Science* publication, Wang and co-authors utilized human cell culture systems, and limited studies were conducted in murine systems. Thus we are in agreement with Dijkstra and Ballingall that the essential conclusions of the *Science* report are not dependent on the murine studies, or the fact that *Wdnm1-like* encodes a long non-coding RNA in humans and the Wdnm1-like protein in mice. However Wang and co-authors used studies in murine systems to further address whether knockdown of Wdnm1-like affected DC differentiation, finally claiming “that *lnc-DC* is vital for DC differentiation in both human and mice”. This strikes one as a very disturbing claim as it implies that in mice this gene locus functions as a long non-coding RNA, when all the available evidence clearly argues against such. This leaves the readers of *Science* with less than the complete and indeed even an obfuscated picture of the *Wdnm1-like *gene. Readers, and one presumes also the reviewers of the *Science* report, were thus unable to fully judge the quality and relevance of the studies that Wang and co-authors conducted in murine systems. As indicated by Dijkstra and Ballingall, Wang and co-authors referring to or renaming murine *Wdnm1-like* as the “ *mouse lnc-DC ortholog*”, without also educating their readers on the protein coding nature of the murine gene, appears a serious disservice to their readers.

 

If Wang and co-authors were aware of the fact that murine *Wdnm1-like* gene was most likely protein-encoding, and whether this was the case or not is unknown at this juncture, they have indeed failed the readers of *Science* in this regard. One would hope to see some explanation from Wang and co-authors on this matter.  The points raised by Dijkstra and Ballingall serve as “food for thought” for all of us on the responsibility of authors to fully inform their readers in regard to the state of current knowledge in respect to the scientific content of their manuscripts.  It is left up to the readers of the paper by Dijkstra and Ballingall, the text of the *Science* report by Wang and co-authors and the comments furnished herein, to reach their individual opinions on this specific matter.

We have read this submission. We believe that we have an appropriate level of expertise to confirm that it is of an acceptable scientific standard.

## References

[1] WuYSmasCM: Wdnm1-like, a new adipokine with a role in MMP-2 activation. *Am J Physiol Endocrinol Metab*.2008;295(1) : 160E205-21510.1152/ajpendo.90316.2008Reference source18492766PMC2493586

[2] BingleCD: Towards defining the complement of mammalian WFDC-domain-containing proteins. *Biochem Soc Trans*.2011;39(5) : 1601393-139710.1042/BST0391393Reference source21936821

[3] WangPXueYHanYLinLWuCXuSJiangZXuJLiuQCaoX: The STAT3-binding long noncoding RNA lnc-DC controls human dendritic cell differentiation. *Science*.2014;344(6181) : 160310-31310.1126/science.1251456Reference source24744378

[4] SharpLZShinodaKOhnoHScheelDWTomodaERuizLHuHWangLPavlovaZGilsanzVKajimuraS: Human BAT possesses molecular signatures that resemble beige/brite cells. *PLoS One*.2012;7(11) : 16010.1371/journal.pone.0049452Reference source23166672PMC3500293

[5] KajimuraSSealePTomaruTErdjument-BromageHCooperMPRuasJLChinSTempstPLazarMASpiegelmanBM: Regulation of the brown and white fat gene programs through a PRDM16/CtBP transcriptional complex. *Genes Dev*.2008;22(10) : 1601397-140910.1101/gad.1666108Reference source18483224PMC2377193

[6] VernochetCPeresSBDavisKEMcDonaldMEQiangLWangHSchererPEFarmerSR: Vernochet, C., et al., C/EBPalpha and the corepressors CtBP1 and CtBP2 regulate repression of select visceral white adipose genes during induction of the brown phenotype in white adipocytes by peroxisome proliferator-activated receptor gamma agonists. *Mol Cell Biol*.2009;29(17) : 1604714-472810.1128/MCB.01899-08Reference source19564408PMC2725706

[7] AdachiWUlanovskyHLiYNormanBDavisJPiatigorskyJ: Serial analysis of gene expression (SAGE) in the rat limbal and central corneal epithelium. *Invest Ophthalmol Vis Sci*.2006;47(9) : 1603801-381010.1167/iovs.06-0216Reference source16936091

# Response

**Published:** 2014-08-27

**Authors:** Johannes M. Dijkstra

**Affiliations:** Fujita Health University, Japan

Dear Dr. Smas and Dr. Ren,

Thank you for your extensive comments, which we embrace as generally positive. We are happy that you approve of how we summarized existing reports on *Wdnm1-like* in rodents. Furthermore, you added some nice insights and discussion points, and provided the readers with additional references that confirm that murine *Wdnm1-like* transcript is highly enriched in white vs. brown adipocytes/adipose tissue. You state correctly that endogenous Wdnm1-like protein has not been reported so far. However, a search of the PeptideAtlas database http://www.peptideatlas.org (Desiere, *et al*. 2006) which includes peptides identified by mass spectrometry from multiple species,  identified a rat peptide encoded by correctly spliced *Wdnm1-like* .  This suggests that the Wdnm1-like protein is present in the rat.  We will include this information in the text after all the referee reports have been received. Whether the style of our manuscript is excessively critical is open to discussion, especially since both our research groups appear to share similar criticisms of the Wang et al. report. However, if you feel that a sentence is excessively critical please send us suggestions for changes  (in a private mail?) and we will follow your lead.

Sincerely,

Johannes M. Dijkstra and Keith T. Ballingall

Reference

Desiere, et al., *"The PeptideAtlas Project"*, Nucleic Acids Research, 2006, 34, D655-D658
